# An analysis of the factors influencing bone health literacy among middle-aged and elderly breast cancer patients undergoing endocrine therapy: a mixed-methods study based on COM-B theory

**DOI:** 10.1186/s12905-026-04326-0

**Published:** 2026-02-11

**Authors:** Ziyue Gai, Nannan Wang, Zichao Gai, Lin Liu, Shan Zhang, Xue Li, Xiaoli Ma, Bo Yan, Baohua Cao

**Affiliations:** 1 Department of Nursing, Air Force Medical University, Xi’an, Shaanxi China; 2 Department of Breast and Thyroid Surgery, Dongying People’s Hospital（Dongying Hospital of Shandong Provincial Hospital Group), Dongying, Shandong China; 3 Shanghai Fifth People’s Hospital, Fudan University, Shanghai, China; 4 Department of Nursing, The 950th Hospital of PLA, Yecheng, Xinjiang China; 5 School of Nursing, Shaanxi University of Chinese Medicine, Xianyang, Shaanxi China; 6 Department of Oncology, The First Affiliated Hospital of Air Force Medical University, Xi’an, Shaanxi China

**Keywords:** Middle-aged and older adults, Breast cancer, Endocrine therapy, Bone health literacy, Family support

## Abstract

**Background:**

Endocrine therapy is a cornerstone in the treatment of breast cancer; however, its prolonged use can exacerbate the deterioration of bone health, particularly in middle-aged and older women, who are already at heightened risk for bone loss due to age-related hormonal changes. Despite the critical role that bone health literacy plays in managing patients′ bone health, there has been a lack of studies examining bone health literacy in middle-aged and older breast cancer patients undergoing endocrine therapy. This study aims to assess the current status of bone health literacy in this demographic and to identify the factors influencing it.

**Methods:**

This study was designed based on the theory of COM-B, employing a convergent design within a mixed-methods theory. For the quantitative component, a convenience sampling method was used to select 261 patients undergoing endocrine therapy to complete a questionnaire survey. For the qualitative component, 11 patients were selected through maximum variation purposive sampling to participate in semi-structured interviews.

**Results:**

The bone health literacy score among middle-aged and older breast cancer patients undergoing endocrine therapy was (43.13 ± 7.23). Quantitative analysis indicates that the primary influencing factors include educational attainment, self-efficacy regarding osteoporosis, and the level of family care. The qualitative research identified four themes and nine sub-themes based on the dimensions of capability, motivation, opportunity, and behavior. Mixed results demonstrate that quantitative findings and qualitative findings complement and corroborate one another.

**Conclusion:**

Middle-aged and older patients undergoing endocrine therapy for breast cancer exhibit low levels of bone health literacy, influenced by a combination of capability, opportunity, motivation, and behavioral factors. This suggests healthcare professionals should prioritize assessing and intervening in bone health literacy among this patient group. Multidimensional interventions targeting these influencing factors can enhance patients’ bone health literacy levels.

## Background

 According to the 2022 data from the International Agency for Research on Cancer (IARC), BC is the second most diagnosed cancer globally, with 2.3 million new cases, representing 11.6% of all cancer diagnoses [1]. It is the leading cancer among women, with middle-aged and elderly women constituting the primary affected demographic. The incidence of BC in this population continues to rise annually, posing a significant and growing threat to their health. Hormone receptor-positive BC accounts for 80% of cases and typically necessitates ET for a period of 5 to 10 years [2, 3]. However, prolonged ET is associated with a range of adverse symptoms, including hot flashes, anxiety, depression, and joint pain. Approximately 30% to 50% of patients undergoing ET experience skeletal muscle symptoms [4]. Among these, middle-aged and older female BC patients are particularly at high risk for osteoporosis. The primary factors contributing to this increased risk can be categorized into three key aspects: First, treatment-related factors: ET, particularly with aromatase inhibitors, induces bone loss at twice the rate observed in healthy postmenopausal women. Approximately 45% of patients experience musculoskeletal disorders [5]. The incidence of fractures is about 18–20% at a 5-year follow-up, with a 1-year mortality rate for hip fractures reaching as high as 33%. Furthermore, around 50% of survivors experience long-term disability as a result of these fractures [6, 7]. Second, age-related factors: For middle-aged and older women, particularly those within 10 years of menopause, the decline in ovarian and muscle function leads to a rapid reduction in estrogen levels, which accelerates bone resorption. This decline in estrogen is associated with an annual bone loss of approximately 2% to 3% [8, 9]. Additionally, middle-aged and older adults often experience chronic diseases and take medications that further contribute to bone loss. Finally, tumor-related factors: BC cells secrete cytokines that disrupt the normal balance of bone metabolism, thereby exacerbating bone loss [10]. This accelerated bone loss increases the risk of secondary osteoporosis and fractures, which significantly impacts patients’ quality of life and medication adherence [4]. Moreover, it may elevate the risk of bone metastasis and mortality, placing substantial health and economic burdens on both patients and the healthcare system [11]. Therefore, considering the factors mentioned above, effective bone health management in middle-aged and older BC patients undergoing ET is crucial for improving their quality of life and overall health outcomes.

Health literacy refers to an individual’s ability to access, comprehend, and utilize basic health information and services to make informed decisions that support and promote their well-being. It is considered the most cost-effective strategy for maintaining health across populations [12]. Building upon this foundation, BHL, a key component of health literacy, encompasses knowledge, behaviors, and skills related to bone health. Currently, a clear, universally accepted definition of BHL is lacking. However, scholars such as Jin CY suggest that BHL includes an individual’s ability to accurately recognize bone health issues, understand related nutritional and exercise principles, identify the primary manifestations of bone health problems, and seek appropriate health services in a timely manner [13]. In this study, BHL refers to the comprehensive capacity of middle-aged and older BC patients undergoing ET to acquire, comprehend, evaluate, and apply bone health-related information, services, and self-management strategies for the prevention and management of bone loss and associated complications. A high level of BHL is essential for effective bone health management in patients. With adequate bone health literacy, these patients are better equipped to identify risk factors, correct detrimental bone health behaviors and lifestyle habits, and proactively engage in interventions. These actions, in turn, can slow the rate of bone loss, reduce the incidence of osteoporosis and fractures, and ultimately improve patients’ quality of survival.

Currently, both domestic and international studies on bone health awareness in BC primarily focus on knowledge of osteoporosis and beliefs related to the condition. However, there remains a gap in specialized research specifically addressing BHL. Several existing studies primarily focus on healthy middle-aged and older women. For example, domestic scholars such as Sun YL conducted a survey of women aged 45–65 years in suburban Shanghai. The results revealed that the BHL level among middle-aged and older women was low, with only 14.5% meeting the standard [14]. Factors influencing this low level included age over 55 years, lower income, and education levels below high school. Similarly, Bailey S and Wang Q found that postmenopausal BC patients exhibit a low level of osteoporosis knowledge. Furthermore, osteoporosis knowledge and beliefs were closely associated with osteoporosis prevention behaviors, such as physical activity and calcium intake, in these patients [15, 16]. Studies have shown that both self-efficacy and family support have a direct promoting effect on the bone health promotion behaviors of women at high risk for osteoporosis. Based on this, we infer that both may also have an important influence on BHL [17]. However, research on BHL in BC patients, particularly middle-aged and older patients undergoing ET, remains limited and requires further investigation.

The COM-B theory was formally proposed by Michie et al. in 2011 [18]. This theory emphasizes that an individual’s behavioral performance depends on the synergistic interaction between three elements: capability, opportunity, and motivation. It provides a robust theoretical foundation for systematically identifying factors influencing health behaviors, particularly aiding in the development of highly targeted behavioral intervention strategies. Within this framework, Capability (C) denotes the physical and psychological skills required for an individual to perform a specific behavior; Opportunity (O) encompasses external conditions such as the physical environment and social support that influence behavior implementation; Motivation (M) includes reflective motivation arising from cognitive appraisal and planning, alongside spontaneous motivation stemming from emotion, impulse, or habit. The COM-B theory has been extensively applied and validated across multiple health behavior research domains, including oral health management, chronic disease prevention and control, and physical activity promotion, demonstrating robust applicability and explanatory power [19–21]. Consequently, the COM-B framework offers compelling theoretical support for investigating BHL among patients, thereby identifying precise targets for clinical intervention design.

ET for BC involves extended treatment cycles. During prolonged treatment, BHL among middle-aged and older patients is subject to complex influences from multidimensional factors including capability, opportunity, and motivation. Given that single quantitative research methods struggle to fully elucidate the underlying mechanisms of potential factors, potentially limiting the interpretative power of findings, this study innovatively employs a convergent mixed-methods approach. By integrating the strengths of quantitative and qualitative research and grounded in the COM-B theory, it systematically explores the relationship between osteoporosis self-efficacy (motivational dimension), family care (opportunity dimension), and bone health knowledge and skills (capacity dimension). It delves into the influencing factors, aiming to provide evidence-based support for developing precise, multi-level bone health intervention strategies at three levels: capacity enhancement, opportunity support, and motivation stimulation.

## Methods

### Design

This study employed a mixed research methodology with a convergent design, comprising both quantitative and qualitative components. Data collection for both the quantitative and qualitative studies was conducted between March 2025 and June 2025.

### Quantitative research

#### Procedure

A convenience sampling method was employed to select 261 BC patients from oncology chemotherapy centers and breast surgery departments in three tertiary hospitals located in Shaanxi and Shandong Provinces, between March 2025 and June 2025. The inclusion criteria were: (1) a pathological diagnosis of BC; (2) age ≥ 45 years; (3) currently undergoing ET; and (4) the ability to communicate effectively. The exclusion criteria included: (1) patients in stage IV; (2) patients with bone metastases; and (3) patients with severe neurological diseases. The sample size was determined using the cross-sectional study formula *N* = P(1-P). Based on a previous study, where *P* = 0.145 [14], and using the values of Z_α/2_ = 1.96 and δ = 0.05, the calculated sample size was *N* = 191. Considering a 20% loss to follow-up, the required minimum sample size was adjusted to 239 cases. Ultimately, the final effective sample size for this study was 261 cases.

### Measurements

#### Bone health literacy questionnaire

The Bone Health Literacy Questionnaire was developed collaboratively by the School of Public Health at Fudan University and the Shanghai Maternal and Child Health Center in 2023 [13]. The questionnaire consists of 40 items across three dimensions: DP (10items), HP (17items), and HC (13items). The items include single-choice, multiple-choice, and scenario-based questions. Each item scores 2 points, with a maximum total score of 80 points. The cut-off value for the overall BHL attainment rate is 54 points, while the cut-off values for the individual dimensions are > 14 points for disease prevention, > 22 points for health promotion, and > 18 points for health care. A higher total score indicates a better level of BHL. In this study, the Cronbach’s alpha coefficient for the scale was 0.771.

#### General information questionnaire

The general information questionnaire included demographic and clinical variables such as age, educational level, occupational status, marital status, monthly income, residence, tumor stage, type of endocrine medication, duration of ET, and whether or not the patient had received radiotherapy.

#### Osteoporosis Self-Efficacy Scale (OSES)

OSES was originally developed by Horan ML and later adapted for use in China by Chen YP [22, 23]. This scale is designed to assess the self-efficacy levels of individuals at high risk for osteoporosis. It consists of two dimensions: OSE-Exercise and OSE-Calcium, with a total of 12 items. The scale has a possible total score range of 0 to 120, with higher scores indicating greater self-efficacy. In this study, the Cronbach’s alpha coefficient for the scale was 0.814.

#### Family APGAR index (APGAR)

The Family APGAR Index (APGAR) was designed by Smilkstein G and later adapted for Chinese populations by Lyu F to assess individuals’ satisfaction with family functioning [24, 25]. The questionnaire comprises five dimensions: adaptability, adulthood, cooperation, affectivity, and intimacy. It is scored using a 3-point Likert scale, with a total score ranging from 0 to 10. The scale’s responses range from 0 to 2, where 0 indicates “seldom” and 2 indicates “often.” Higher scores reflect better family functioning. In this study, Cronbach’s alpha coefficient for the scale was 0.896.

### Data collection

To ensure consistency in the data collection process, researchers across all centers underwent standardized research protocol and ethics training. During recruitment, researchers screened patients meeting inclusion criteria daily from inpatient or outpatient systems, conducting face-to-face interactions to distribute standardized information booklets and providing detailed explanations of the study. Following written informed consent, participants could choose to complete the questionnaire either offline or online based on their circumstances: offline involved completing, verifying, and collecting paper questionnaires on-site; while online questionnaires were distributed via the “Wenjuan Xing” platform, requiring all questions to be answered with each WeChat ID permitted only one submission. Responses taking ≤ 8 min to complete were excluded. Throughout the study, regular meetings were held alongside randomized spot checks of data collection procedures across centers to ensure operational standardization and data quality.

### Data analysis

Data were analyzed using SPSS version 26.0. Categorical data are presented as frequencies and percentages, while continuous variables are expressed as mean±standard deviation. Comparisons between groups were conducted using the independent samples t-test and one-way analysis of variance (ANOVA). Multiple linear regression analysis was performed with the total BHL score as the dependent variable and independent variables selected based on statistically significant differences (*P* < 0.05) from the correlation analysis and one-way ANOVA. The significance level was set at α = 0.05.

### Qualitative research

#### Procedure

Purposive sampling was employed based on the principle of maximum variation to select patients from the breast surgery outpatient clinic and inpatient wards who were undergoing ET and met the inclusion and exclusion criteria (consistent with the quantitative study). Participants were chosen to represent diverse age groups, educational backgrounds, occupational statuses, and income levels. Recruitment and interviews were conducted by one uniformly trained nursing graduate student with experience in qualitative research. The sample size was determined according to the principle of data saturation in qualitative research, whereby recruitment ceased when consecutive interviews no longer yielded new information or themes.

### Measurement

Based on the COM-B theory and the research objectives, a preliminary interview guide was developed. This outline was revised after conducting interviews with 2–3 patients, incorporating expert guidance and feedback. See Table 1.

**Table 1:** Interview guide

Capability	• What is your understanding of the potential bone health issues associated with ET for BC? • What measures have you taken to prevent or address these bone health issues? From which sources did you obtain this information?
Opportunity	• What are the main difficulties or obstacles you encounter when learning about and practicing bone health management? • What relevant support has your family, friends, or healthcare team provided? In what specific ways?
Motivation	• How confident are you in your ability to adhere to these bone-protecting measures (such as long-term calcium supplementation and regular exercise)? Why? • How confident are you in maintaining these bone-protecting measures (such as long-term calcium supplementation and regular exercise)? Among the many demands of your daily life (such as medical treatment, family commitments, and work), how important do you consider adhering to bone health management? How much effort are you willing to dedicate to it?
Behavior	• During your treatment, did you experience any bone pain or fractures? How were these issues addressed at the time? • How is your intake of calcium and vitamin D-rich foods (such as milk and fish) in your diet? Could you provide some examples? • How often do you exercise? What types of exercise do you engage in, and how frequently?

### Data collection

The qualitative study employed semi-structured interviews. Prior to the interviews, patients were briefed on the purpose of the study and the necessary precautions to ensure consistency in the interview topics and encourage patient cooperation. Upon obtaining informed consent, patients were selected for face-to-face interviews, which took place in a comfortable and quiet environment. The entire interview process was audio-recorded, with each session lasting between 20 and 30 min. The interviews followed a structured outline, though communication techniques were flexibly applied to facilitate questioning. Throughout, efforts were made to respect and protect the privacy of the participants. The recorded data was transcribed into text within 24 h of the interview to ensure timely analysis.

### Data analysis

The transcribed texts were stored and managed using NVivo 20.0 software, and the data were analyzed using content analysis [26]. Data analysis was conducted independently by two researchers. In cases of disagreement, the research team held discussions to reach a consensus on the final results.

### Integration of mixed research

This study, grounded in the COM-B theory, presents a visualized comparison of quantitative and qualitative research findings through a tabular format, integrating the results of both methodologies. The specific integration methods are as follows: (1) Convergence: quantitative research findings align with qualitative research findings; (2) Complementarity: results from both phases mutually supplement each other; (3) Silence: when only quantitative or qualitative research findings are incorporated; (4) Inconsistency: when quantitative research findings and qualitative research findings are at odds.

## Results

### Quantitative results

#### The demographics characters and univariate analysis of participants

In this study, 280 questionnaires were distributed, and 261 valid questionnaires were returned, yielding a valid response rate of 93.21%. Of the 261 valid questionnaires, 203 (77.8%) were paper-based and 58 (22.2%) were completed electronically. Nineteen distributed questionnaires were excluded for the following reasons: 11 due to non-return or participant withdrawal, 5 due to incomplete data, and 3 due to invalid response patterns (e.g., patterned responses or logical inconsistencies). A total of 261 patients were included in the analysis, with a mean age of 52.06 ± 6.30 years. The results of the univariate analysis revealed significant differences in BHL among patients based on factors such as age, educational level, occupational status, monthly income, residence, and the use of endocrine medications (*P* < 0.05). More detailed information is in Table 2.

**Table 2:** Demographics characters and univariate analysis of participants (*N*=261)

variables	Number (％)	BHL score (Mean ± SD)	*t/F*	*P*
Age(years)			4.875	＜0.001
45-55	152（58.2）	44.92±6.59		
≥56	109（41.8）	40.66±7.38		
Educational level			69.263	＜0.001
Junior secondary and less	93（35.6）	35.67±5.66		
High school/junior college	101（38.7）	40.53±5.33		
Bachelor and more	67（25.7）	42.68±5.84		
Occupational status			24.083	＜0.001
Be employed	105（40.2）	45.43±6.74		
Separation/retirement	65（24.9）	44.88±7.23		
Full-time at home	91（34.9）	39.23±6.12		
Marital status				
Married	246(94.3)	43.29±7.19		
Unmarried	5(1.9)	43.40±5.27		
divorced/widowed	10(3.8)	39.10±8.41		
Monthly income，RMB				
＜3000	61（23.4）	39.05±5.53	20.508	＜0.001
3000-5000	101（38.7）	42.75±7.78		
＞5000	99（37.9）	46.03±6.25		
Residence			7.536	＜0.001
City	178（68.2）	45.22±6.72		
Village	83（31.8）	38.65±6.21		
TNM stage			1.65	0.194
Stage I	75（28.7）	44.20±6.89		
Stage Ⅱ	115（44.1）	42.30±7.25		
Stage Ⅲ	71（27.2）	43.37±7.47		
Duration of endocrine therapy (months)			1.878	0.134
≤12	83（31.8）	42.43±6.83		
13-24	59（22.6）	42.58±7.31		
25-36	38（14.6）	45.61±6.61		
＞36	81（31）	43.09±7.71		
Endocrine drugs			2.634	0.035
Tamoxifen	36（13.8）	41.94±5.79		
Exemestane	122（46.7）	42.63±7.58		
Toremifene	34（13）	46.74±6.57		
Anastrozole	36（13.8）	43.19±7.03		
Letrozole	33（12.6）	42.48±7.46		
Radiotherapy			-1.798	0.086
Yes	241（92.3）	42.90±7.19		
No	20（7.7）	45.95±7.31		

#### The scores on BHL, OSES and APGAR of participants

The BHL score of patients was 43.13±7.23, with an attainment rate of 13.41%. The scores across the dimensions, in descending order, were as follows: DP, HC, and HP. The APGAR score was 6.28±2.18, and OSES score was 81.03±9.54. The mean scores for each dimension of OSES, in descending order, were calcium intake efficacy and exercise efficacy. See Table 3.

**Table 3:** The score of BHL, OSES and APGAR(*n* = 261)

variables	items	Score (Mean ± SD)	Items mean score	attainment rate(%)
BHL	40	43.13 ± 7.23	1.08 ± 0.18	13.41
DP	10	14.89 ± 2.37	1.49 ± 0.24	23.37
HP	17	13.15 ± 3.56	0.77 ± 0.21	3.07
HC	13	15.09 ± 3.38	1.16 ± 0.26	18.77
OSES	12	81.03 ± 9.54	6.75 ± 0.80	—
OSE-Exercise	6	37.61 ± 7.96	6.27 ± 1.33	—
OSE-Calcium	6	43.42 ± 5.59	7.24 ± 0.93	—
APGAR	5	6.28 ± 2.18	1.61 ± 0.49	—

#### The pearson correlations between BHL, OSES and APGAR of participants

Pearson’s correlation analysis revealed a positive correlation between the total BHL score and its dimension scores with both OSES (*r* = 0.345, *P* < 0.001) and APGAR (*r* = 0.720, *P* < 0.001) in patients.

#### The multiple linear regression of factors influencing BHL

The multiple linear regression of factors influencing BHLMultiple linear regression analysis was conducted using the total BHL score of patients as the dependent variable. Independent variables included those that were statistically significant (*P* < 0.05) in the univariate analyses, as well as the total OSES score and the total APGAR. The results of multicollinearity analysis indicated that *VIF* was less than 5, suggesting no serious covariance among the independent variables. The analysis revealed that higher education (university level and above, with junior high school and below as the reference), OSES (original value input), and APGAR (original value input) were the main factors influencing BHL, explaining 65.5% of the total variance. See Table 4.

**Table 4:** Multiple linear regression analysis of BHL(*N* = 261)

Variables	B	S.E.	β	t	*p*	95%CI	VIF
(Constant)	22.950	3.219	-	7.128	< 0.001	16.608 ~ 29.291	-
Bachelor and more	5.044	1.002	0.305	5.033	< 0.001	3.070 ~ 7.019	2.773
OSES	0.117	0.030	0.154	3.921	< 0.001	0.058 ~ 0.176	1.164
APGAR	1.814	0.142	0.547	12.756	< 0.001	1.534 ~ 2.095	1.382

*R*^2^=0.673，adjusted *R*^2^=0.0.655，F=36.198，*P*<0.001

### Qualitative results

#### The demographics characters of participants

A total of 11 middle-aged and older patients undergoing ET for BC were included, with a mean age of (50.91 ± 5.26) years. They were designated as P1-P11. See Table 5.

**Table 5:** Demographics characters of participants (*N* = 11)

ID	Age	Educational level	Occupational status	Monthly income, RMB	Residence
P1	54	High school/junior college	Be employed	3000–5000	Village
P2	45	Bachelor and more	Be employed	>5000	City
P3	55	Junior secondary and less	Full-time at home	<3000	Village
P4	46	Bachelor and more	Be employed	>5000	City
P5	53	Bachelor and more	Be employed	3000–5000	City
P6	50	Junior secondary and less	Full-time at home	<3000	Village
P7	47	High school/junior college	Be employed	3000–5000	City
P8	46	High school/junior college	Be employed	3000–5000	City
P9	50	High school/junior college	Be employed	3000–5000	Village
P10	63	High school/junior college	Separation/retirement	<3000	City
P11	51	Bachelor and more	Be employed	3000–5000	City

### Themes from the qualitative content analysis

#### Theme 1: bone health management capability

##### Subtheme 1.1 Attribution bias in bone health issues 

Patients commonly exhibit cognitive biases regarding the bone health risks associated with ET, frequently attributing skeletal symptoms to ageing or fatigue. This reflects a systematic lack of disease-specific knowledge.

*" I’m not entirely clear about the bone health risks related to endocrine therapy. I do occasionally have back pain—likely due to fatigue*,* but it doesn’t seem serious. At my age*,* it’s often hard to trace where such discomfort comes from.“(P6)*.

*" I haven’t paid close attention to these bone-related issues from endocrine therapy. I continue with my daily work as usual. Sometimes there’s mild discomfort in my lower legs*,* but it passes quickly. I consider that quite normal and not something to be concerned about.“(P7)*.

##### Subtheme 1.2 Inadequate awareness of the severity of bone loss risk 

Although patients may be aware of osteoporosis, they generally underestimate the severity and urgency of its progression, failing to develop sufficient awareness of the health threat it poses. They tend to regard bone health issues as either manageable overtime or as minor accompanying conditions.

*" I was already diagnosed with osteoporosis initially*,* but it hasn’t significantly impacted my daily life.“(P9)*.

#### Theme 2: opportunities in bone health management

##### Subtheme 2.1 the squeeze on bone health management

 Due to Family Responsibilities Patients bearing primary family caregiving responsibilities experience a severe marginalization of personal health management in their allocation of time and energy, creating a structural impediment.

*“My daily diet is relatively monotonous — mainly regular home meals*,* without any intentional addition of calcium-rich foods. Besides*,* with having to care for the grandchildren*,* it’s just not feasible to manage everything at once.“(P3)*.

*“My husband had a stroke some time ago*,* and the children are all at work and not at home. I’m mainly responsible for cooking for her*,* administering her medication*,* and taking our grandson to and from school. I simply don’t have the time or energy.“(P10)*.

##### Subtheme 2.2 inadequate medical support and the complementary role of peer support

 Due to constraints imposed by the pace of clinical practice, healthcare institutions lack systematic preventive guidance. Consequently, the sharing of experiences and emotional support among fellow patients becomes a vital channel for supplementing information and reinforcing behavioral change.

*“Sometimes doctors might be too busy to explain everything in detail*,* so they tend to focus on delivering the most critical information.“(P2)*.

*“Currently*,* doctors rarely provide supplementary guidance — such as how to prevent future problems or what side effects might arise from the medication. I can’t recall any doctor ever mentioning these aspects to me.“(P4)*.

*“We patients often exchange views among ourselves about which calcium supplement might work better.“(P11)*.

#### Theme 3: motivation for bone health management

##### Subtheme 3.1 insufficient sense of self-efficacy

Patients commonly exhibit a lack of confidence in their ability to adhere to regular exercise and healthy behaviors. Their sense of self-efficacy is readily influenced by personal circumstances, environmental changes, and occasional discomfort. This diminished self-efficacy significantly undermines their motivation and resolve to initiate and sustain health management practices.

*“I’ve never been much of an exerciser. When you feel a bit low on energy*,* you just don’t have the drive. And in winter*,* wearing all those layers of clothing*,* you feel even less motivated to move around.“(P3)*.

*“I’ve also signed up for yoga classes to keep fit*,* though occasionally I skip them if I’m feeling unwell. And during hot weather*,* I tend to neglect them somewhat. “(P5)*.

##### Subtheme 3.2 anxiety avoidance tendencies in processing bone health information

When confronted with specialized bone health information, some patients experience “information overload” due to psychological stress. They subsequently adopt avoidance strategies to alleviate short-term anxiety, yet this further restricts cognitive enhancement and behavioral decision-making.

*“After reading through so much health-related popular science information*,* I feel both mentally burdened and emotionally exhausted.“(P6)*.

*“I reckon people are just muddle-headed anyway*,* and I’ve got a pretty easy-going attitude about it—I don’t dwell on things too much.“(P8)*.

#### Theme 4: bone health management practices

##### Subtheme 4.1 proactive information acquisition and multi-channel learning

Patients actively utilize diverse channels such as books, short videos, and medication leaflets to supplement and filter knowledge concerning bone health, demonstrating autonomy and adaptability in their information-seeking behavior.

*“In my spare time*,* I read books. Some doctors also run science-popularization livestreams or post videos on Douyin and Kuai Shou — I’ve learned quite a bit from those.“(P1)*.

*“I watched a few short videos online*,* and the medication leaflet also listed side effects*,* including possible adverse reactions related to endocrine therapy.” (P11)*.

##### Subtheme 4.2 structured lifestyle management

The patient is able to integrate various health-promoting behaviors, such as daily diet and nutritional supplementation, into a systematic and planned personal health management program, demonstrating sound self-management capabilities.

*“I have developed a routine of getting regular sun exposure*,* drinking milk*,* eating plenty of vegetables*,* and maintaining a balanced diet*,* while also taking calcium and vitamin supplements daily.“(P2)*.

##### Subtheme 4.3 holistic mind-body adaptation

Some patients focus not only on physical health but also actively seek and practice integrated approaches—including traditional exercises and psychological learning—to achieve synergistic enhancement and positive adaptation of both physical and mental states.

*“I practice traditional exercises like Vajra Gong through online platforms and also listen to some free psychology courses. These have given me a lot of support.“(P2)*.

### Integrated results

The findings from quantitative and qualitative research complement and corroborate one another across the four core dimensions of capability, motivation, opportunity, and behavior. Further integrated analysis reveals that educational level, self-efficacy, social support, cognitive bias, family role stress, information anxiety, and active health behavior strategies collectively constitute key factors influencing BHL. See Table 6.

**Table 6:** Joint display of integrated quantitative and qualitative research results

Theory	Quantitative research	Qualitative research	Integration
Capability	Higher education qualifications are positively correlated with BHL (*β* = 0.305, *P* < 0.001)	Insufficient bone health management cognition λ Inadequate Awareness of Bone Health Issues λ Diminished Perception of Bone Loss Risk	Convergence: The qualitative finding inversely reflects the quantitative conclusion that higher education enhances capabilities.
Opportunity	Family care levels are positively correlated with BHL (*r* = 0.720, *P* < 0.001)	Constraints and Enablers for Bone Health Management Opportunities λ The Squeeze on Bone Health Management λ Inadequate Medical Support and the Complementary Role of Peer Support	Complementarity: Quantitative research supports the role of family care, whilst qualitative studies reveal its function through alleviating role-related stress and supplementing inadequate medical support, thereby enriching the conceptualization of the “opportunity” dimension.
Motivation	Self-efficacy regarding osteoporosis is positively correlated with BHL (*r* = 0.345, *P* < 0.001)	Weak motivation for bone health management λ Insufficient Sense of Self-Efficacy λ Anxiety Avoidance Tendencies in Processing Bone Health Information	Convergence+Complementarity: Qualitative “insufficient efficacy” and quantitative findings jointly corroborate the central role of efficacy; “diminished risk perception” and “information anxiety avoidance”complementarily expand the affective and cognitive dimensions within motivation.
Behavior	—	Proactive Health Behavior Strategies λ Proactive Information Acquisition and Multi-Channel Learning λ Structured Lifestyle Management λ Holistic Mind-Body Adaptation	Silence: Qualitative findings clarify the specific behavioral strategies individuals may adopt when endowed with the requisite capability, opportunity, and motivation.

## Discussion

In this study, the BHL score among middle-aged and older BC patients on ET was 43.13 ± 7.23, with a compliance rate of only 13.41%—both significantly lower than values reported for healthy middle-aged and older women [14]. This discrepancy may stem from geographical, socioeconomic, and population differences. The results indicate that while patients have basic bone health knowledge, their understanding remains fragmented, especially in the HP dimension (mean score 0.77 ± 0.21; attainment rate 3.07%). Therefore, healthcare professionals should take improving patients’ BHL as a core objective. Assessments can be used to identify cognitive and behavioral gaps in areas such as “health promotion,” which can then be addressed through targeted educational interventions and behavioral guidance, thereby promoting the systematic development of bone health literacy.

Mixed-method research reveals that patients’ BHL is influenced by multiple factors, including education, self-efficacy, family support, family role stress, information anxiety, and proactive health behavior strategies. In terms of capability, the study found that educational attainment is a significant predictor of BHL, consistent with findings from Wei C W and other researchers [27]. More educated patients demonstrate stronger abilities in accessing, comprehending, and applying bone health information, enabling them to better recognize treatment-related risks and take preventive action. Qualitative research further reveals that patients with lower educational attainment are prone to attribution bias and inadequate risk perception. This suggests that education shapes not only information processing but also underlying cognitive patterns of illness attribution and risk perception. Therefore, healthcare professionals are advised to use visual tools such as short videos and infographics in place of text-heavy materials, while actively correcting cognitive biases and enhancing risk awareness. Supplementing these with immediate consultation channels can improve information accessibility and understanding, thereby strengthening patients’ capacity for bone health management.

In terms of opportunity, a significant positive correlation was found between family support and BHL, consistent with the findings of Zhang Wei et al. [17]. Qualitative research further illuminates the complexity of social support systems: on the one hand, families and peers respectively provide behavioral support and emotional information support. Robust family and peer support can exert a positive influence on patients’ treatment and recovery [28–30]. On the other hand, influenced by traditional family values, middle-aged and older adults predominantly assume the role of primary carers [31]. Many patients marginalize their own health management due to caregiving responsibilities, while inadequate preventive guidance during outpatient consultations further hinders the implementation of systematic support. This suggests that opportunity is shaped not by support alone, but by a dynamic interplay between social support, peer networks, and individual role obligations. Therefore, a multi-level “hospital-family-community” support system is recommended. This could include developing self-help tools, facilitating peer support groups, delivering family education, and providing community respite services to structurally enhance patients’ opportunities for bone health management.

In terms of motivation, this study identified that self-efficacy regarding osteoporosis among patients constitutes a key determinant of their BHL. This finding is broadly consistent with the research outcomes of Nver et al. [32]. Self-efficacy regarding osteoporosis refers to an individual’s assessment of their confidence in executing health behaviors related to osteoporosis prevention (such as regular exercise and calcium intake) [23]. Its level directly influences patients’ health behavior choices and long-term self-management capabilities [33]. Patients with high self-efficacy not only proactively adopt health behaviors like calcium supplementation and regular exercise but also demonstrate sustained adherence over time. However, qualitative insights reveal a contradictory psychological process: patients often experience anxiety from excessive health information, yet simultaneously employ avoidance strategies to relieve short-term distress. This “anxiety-avoidance-cognitive deficiency” cycle, intensified by the inherent stress of cancer diagnosis and treatment, creates a complex motivational barrier. It suggests that self-efficacy encompasses not only behavioral confidence but also emotional regulation and cognitive adaptation during health information processing. Therefore, healthcare professionals could enhance self-efficacy through cognitive-behavioral techniques, while adopting a phased, stepwise approach to information delivery to prevent overload and mitigate anxiety.

In terms of behavior, proactive health behaviors enhance patients’ motivation for bone health management, thereby contributing to improved BHL. Proactive health behaviors refer to the measures and actions taken by individuals to maintain and enhance their own health, emphasizing the active participation and positive engagement demonstrated by individuals throughout the health management process [34]. Qualitative findings show that patients who actively seek information and adopt preventive measures tend to experience better bone health outcomes. Supporting this, research on middle-aged and older adults confirms that proactive health behaviors positively predict health literacy levels [35]. Thus, encouraging such behaviors can effectively promote engagement in bone-health learning and the formation of sustainable habits. Accordingly, healthcare professionals should adopt a multi-strategy approach that combines education on daily management, phased goal setting with feedback, and contextualized guidance. This will help instill in patients a sense of being the “primary custodian” of their own health, thereby fostering lasting proactive behaviors and enhancing BHL.

## Conclusion

This study, guided by the COM‑B theoretical framework, employed a mixed‑methods approach to systematically analyze the current status and influencing factors of bone health literacy (BHL) among middle‑aged and older breast cancer patients undergoing endocrine therapy. Quantitative results indicate that BHL levels in this population are generally low, with osteoporosis‑related self‑efficacy, family support, and educational level identified as key influencing factors. Qualitative research further elucidates the complex mechanisms through which social support systems enhance BHL and identifies other critical factors such as cognitive bias, family role stress, information anxiety, and proactive health behavior strategies. The quantitative and qualitative findings corroborate and complement each other across the four dimensions of capability, motivation, opportunity, and behavior, offering a more systematic and nuanced perspective for understanding the barriers and facilitators to bone health management in this population. Therefore, healthcare professionals are advised to develop systematic, multi‑level, and actionable intervention strategies targeting these key factors, in order to systemically enhance patients’ BHL, promote the adoption and maintenance of healthy bone‑related behaviors, and ultimately improve their long‑term quality of life and prognosis.

### Strengths and limitations

This study employed a mixed-methods approach to examine bone health literacy (BHL) and its influencing factors among middle‑aged and older breast cancer patients undergoing endocrine therapy, offering insights for individualized bone health management in clinical practice. However, several limitations should be noted. First, the cross‑sectional design limits causal inference and longitudinal observation of BHL changes. Second, convenience sampling from tertiary hospitals may affect the representativeness and generalizability of findings, with urban‑rural disparities not thoroughly explored. Third, the BHL assessment tool was originally developed for healthy middle‑aged and older women and may not fully capture the specific concerns of breast cancer patients on endocrine therapy. Finally, despite being theory‑guided, not all potential influencing variables were included. Future research could adopt longitudinal designs, expand sampling to diverse populations, develop breast cancer‑specific BHL tools, and incorporate a wider range of variables to improve scientific rigor.

## Data Availability

The data that support the findings of this study are available from the corresponding author upon reasonable request.

## Abbreviations

BC: Breast cancer
ET: Endocrine therapy
BHL: Bone health literacy
DP: Disease prevention
HP: Health promotion
HC: Health care
OSES: Osteoporosis self-efficacy scale
OSE-Calcium: Osteoporosis self efficacy-calcium
OSE-Exercise: Osteoporosis self efficacy-exercise
APGAR: Family APGAR index
COM-B: Capability, opportunity, and motivation-behavior
VIF: Variance inflation factor
